# The Influence of REE Steel Modification on the Microstructure and Mechanical Characteristics Using Fractographic Analyses

**DOI:** 10.3390/ma18235408

**Published:** 2025-11-30

**Authors:** Robert Pała, Piotr Furmańczyk

**Affiliations:** Department of Machine Design, Faculty of Mechatronics and Mechanical Engineering, Kielce University of Technology, Av. 1000-an. of Polish State, 7, 25-314 Kielce, Poland; pfurmanczyk@tu.kielce.pl

**Keywords:** microstructure, fracture toughness, REE modification, REM modification, fractography, microroughness, fracture mechanics

## Abstract

Improving the operational parameters of machinery necessitates the use of materials with higher mechanical characteristics. Strength characteristics, particularly fracture toughness, are strongly linked to the material’s microstructure. This article presents the results of a study examining the effect of microstructure on the mechanical properties and fracture toughness of G17CrMo5-5 cast steel in its basic and rare-earth modified variants. The addition of rare-earth elements (REEs) to the melt resulted in a reduction and homogenization in grain size, as well as a reduction in the size and shape of non-metallic inclusions. For modified cast steel, there were no grains with a chord size above 120 μm and inclusions with a diameter above 5.5 μm. Changes in the microstructure of modified cast steel resulted in a slight increase in strength properties. It significantly increased the fracture toughness: for unmodified cast steel at a temperature of −20 °C, the fracture toughness increased from 94 kN/m to 416 kN/m for modified cast steel. Fracture fractographic analysis using non-contact microroughness measurement techniques or measuring the width of the stretch zone allowed for the calculation of fracture toughness without the need for a conventional test. Fracture toughness calculated based on fractographic analysis can be determined for brittle fracture and brittle fracture preceded by plastic growth. Numerical simulations of the loading of specimens tested for fracture toughness allowed us to determine the effect of the REE steel modification on the stress field distribution ahead of the crack front. The modification resulted in a change in the opening stress distribution and the location of its maximum at each temperature. The use of REE modification is an effective approach for homogenizing the microstructure and increasing the fracture toughness of cast steel, especially when the material operates at temperatures in the interval of the fracture mechanism change.

## 1. Introduction

Growing demands on the operational parameters of heavy machinery pose new challenges for materials engineering. These include new materials that influence strength parameters, impact strength, toughness, and fracture toughness. Improvements to the operational results can be achieved in two ways: by replacing previous materials, which is costly, or by modifying the materials currently presented, which determines the application and solution [1]. This challenge can also be applied to modern metallurgical technologies found in earth metals [2]. In the literature, the terms “rare earth elements” (REEs) and “rare earth metals” (REMs) are often used interchangeably.

The mechanical properties of cast steel and steel can be successfully modified by adding rare earth elements and an appropriately selected heat treatment. Skołek et al. demonstrated that this method can be used to obtain a cast steel microstructure characterized by high tensile strength [3,4]. The authors emphasize that the microstructure largely depends on the heat treatment parameters used.

Rare earth elements, such as cerium, lanthanum, and yttrium, play a significant role in the modification and refining of steel. Their presence improves the mechanical and operational properties. In low-alloy steels, the addition of cerium and lanthanum modifies sulfide inclusions, transforming elongated MnS particles into spherical ones, which leads to the increased plasticity and impact strength of the steel [5]. In heat-resistant steels used for turbine and power boiler components, cerium stabilizes non-metallic inclusions and limits intergranular cracking, which translates into increased creep resistance at high temperatures [6]. Rare earth elements are also used in manganese (Hadfield) cast steel, where their addition promotes austenite grain refinement and uniform inclusion distribution. This increases the impact strength and abrasion resistance, which is important in components operating under intense wear conditions, such as crusher jaws or mill casings [7]. Additionally, cerium and yttrium bind sulfur and phosphorus, reducing the risk of hot cracks in welds, improving the weldability of structural steel and facilitating the formation of durable joints in large castings [8].

The introduction of small amounts of rare earth elements, most commonly cerium (Ce) and lanthanum (La), has proven to be an effective way to improve critical material parameters such as impact strength and fracture toughness. The main mechanism of action of REEs in liquid steel is their high affinity for trace elements, particularly sulfur and oxygen. In traditional steel, sulfur forms elongate manganese sulfide (MnS) inclusions with manganese (Mn), which align along grain boundaries, creating preferential paths for crack initiation and propagation [9,10]. The addition of REEs changes this situation; these elements bind sulfur, forming stable, high-melting sulfides that crystallize as fine, spherical particles. This change in the morphology of the inclusions significantly reduces their detrimental effect on the material’s continuity [11].

After solidification, the beneficial effects of inclusion modification are complemented by additional microstructural mechanisms. The spherical REE inclusions act as effective nucleation centers for growing grains, leading to refinement of the casting structure [9,12]. Fine grain size is, in turn, a key factor in increasing both the strength and impact toughness of the material.

A number of scientific studies have demonstrated the strong dependence of the microstructure on the use of rare earth elements, which directly affects the strength properties, fracture toughness, and impact strength. In the study by Zhao et al. [13], the thermal and plastic treatment of microalloyed cast steel resulted in a nearly elevenfold increase in impact strength while simultaneously improving the strength characteristics. A similar effect of REE modification on steel was demonstrated by Zhao et al. in another publication [14]. The addition of REEs refined and homogenized the microstructure, increased the tensile strength and elongation, and changed the crack morphology.

Studies by Chaus [15] on R6M5K5 and R6M5 steels demonstrated that modification changed the chemical composition, morphology, and carbide distribution. The relationship between structural parameters and mechanical property characteristics was also determined. In turn, the publication by Xu et al. demonstrated that the addition of yttrium (Y) improved the microstructure, Vickers hardness, tensile strength, impact strength, and fatigue properties of bearing steel. The results show that yttrium can refine and spheroidize cementite, making its distribution more uniform [16].

This article describes the influence of the microstructure of G17CrMo5-5 cast steel on its strength and fracture toughness characteristics. Research and analysis were conducted on two melt variants: the unmodified (UM) and the REE-modified (M). Stress–strain curves and strength characteristics were determined. Fracture toughness was assessed using ASTM, and a method based on microroughness measurement was tested.

Classical tests were used to determine the fracture toughness in the works cited above. The rapid development of non-contact measurement techniques allows for their use in determining fracture toughness. The use of an optical profilometer in fracture surface analysis enabled the measurement of microroughness. Fracture toughness was determined based on the guidelines provided by R.O. Ritchie and A.W. Thompson [17].

For cases where the brittle fracture mechanism dominated, limiting the fracture microroughness measurement method, a method based on measuring the width of the stretch zone, was used. The traditional method, based on discrete points, is problematic. This causes difficulties in interpreting the zone boundaries and leads to measurement uncertainty due to the irregularity of the stretch zone, or even its local disappearances. A modification was proposed, which involves measuring the area of the stretch zone instead of its width at individual points. For this purpose, the image analysis program ImageJ2x was used.

Fracture toughness values obtained according to the ASTM E1820 standard [18] were compared with a method based on measuring microroughness and stretch zone width. Changes in fracture toughness were explained based on the microstructure.

The results of numerical calculations indicate differences in the stress field distributions ahead of the crack front for the analyzed cast steel melts.

In summary, this article presents a comprehensive approach to assessing the effect of modifications to REE G17CrMo5-5 steel on the microstructure, strength properties, and fracture toughness. Changes in microstructural morphology, including the frequency and shape of grains and inclusions of specific sizes, were considered. Classical strength and fracture toughness tests were conducted in the temperature range of 20 °C to −80 °C. This temperature range ensured that the fracture toughness tests encompassed a range of fracture mechanisms, from ductile fracture through mixed fracture (brittle fracture preceded by plastic growth), to purely brittle fracture. Based on the fracture toughness test results, innovative approaches were tested to calculate the fracture toughness based on fracture fractographic analysis. The presented methods allow for the calculation of fracture toughness within the fracture zone: brittle fracture preceded by plastic growth, and brittle fracture, without the need for a classic fracture toughness test. The applied research methodology allows for the use of fractographic analyses, defining new fracture zones for quantitative fracture toughness assessment.

## 2. Materials and Methods

The first stage of the study involved microstructure analyses of G17CrMo5-5 steel in its unmodified and rare earth element-modified states. Grain size and inclusion fractions were determined from metallographic microsections after their preparation by polishing and etching with Nital.

In the second stage, mechanical tests were conducted at temperatures ranging from −80 °C to 20 °C using liquid nitrogen vapor as the refrigerant. Loading was performed on a UTS Zwick-100 machine with a screw drive (ZwickRoell GmbH & Co KG August-Nagel-Str. 11 89079 Ulm Germany). Uniaxial tensile testing was performed on specimens with a diameter of 5 mm and a measurement base of 25 mm, in accordance with the recommendations of standards [19,20]. Fracture toughness was determined on specimens in the shape of a prismatic beam with a single-sided SENB (Single Edge Notch Bending) notch, from the bottom of which a fatigue crack was introduced. The combined depth of the notch and fatigue crack was ~0.5 W. The dimensions of the tested specimens were thickness—12 mm, width—24 mm, and support spacing—96 mm.

In the third stage, fractographic analyses were conducted based on microroughness measurements in the crack growth area. A Talysurf CCI Lite optical profilometer was used for these measurements. The proposed approach enabled the determination of fracture toughness based on the measured microroughness. SEM images (JEOL 7100F) of the fracture surface structure revealed areas of ductile, brittle, and brittle fractures.

The fourth stage involved assessing the effect of inclusion shape on the stress field distributions ahead of the crack front. Numerical calculations were performed for two melt variants tested at temperatures of 20, −20, and −60 °C.

## 3. Results

The cast steel G17CrMo5-5 belongs to the group of low-alloy cast steels and is designed for use at elevated temperatures, around 530 °C. Due to its good strength properties, it is used for machine parts operating under high mechanical loads combined with thermal impact. It is used, among other things, in the construction of steam turbine valve chambers. To enhance the properties of steel, particularly impact strength and fracture toughness, additional metallurgical treatments are increasingly being used, including the introduction of microadditives. Modification involves the introduction of modifiers during the tapping of the steel into the ladle, usually in amounts no greater than 0.1%.

**Figure 1:**
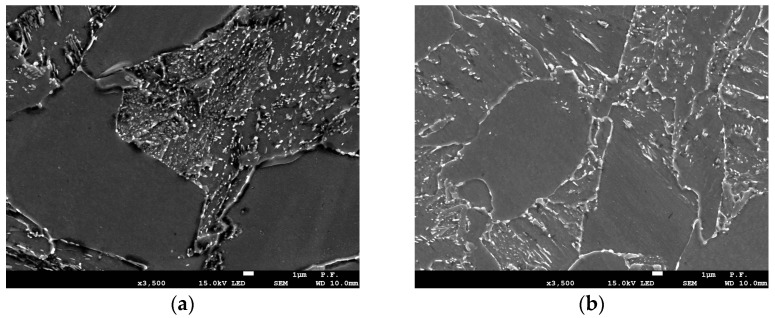
Microstructure of G17CrMo5-5 cast steel: (**a**) unmodified; (**b**) modified (etched with Nital).

**Figure 2:**
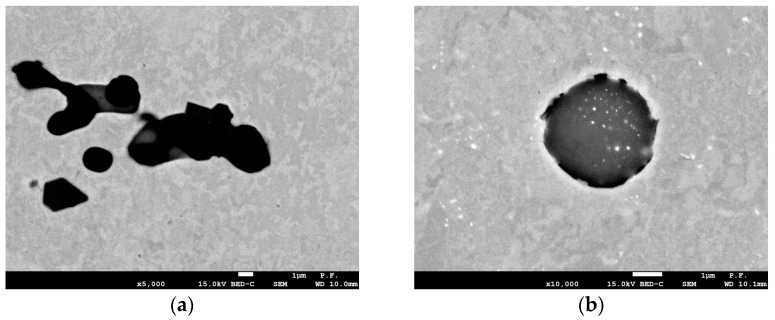
Shape of non-metallic inclusions in cast steel: (**a**) UM; (**b**) M.

Cast steel with the chemical composition given in Table 1 was tested in two variants: unmodified and modified. The composition of the unmodified cast steel was consistent with [21]. Modification was performed by adding mischmetal with the composition of 49.8% Ce, 21.8% La, 17.1% Nd, 5.5% Pr, 5.35% during tapping into the ladle. The modification of REEs resulted in changes in the microstructural of the cast steel-grain refinement (Figure 1) and spheroidization of non-metallic inclusions (Figure 2).

**Figure 3:**
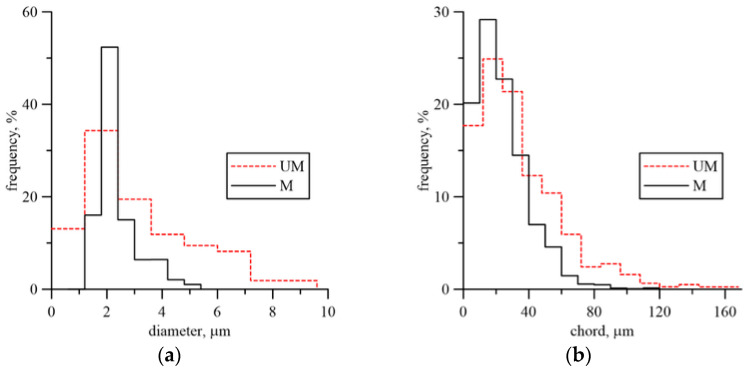
Microstructural features of UM and M cast steel: (**a**) diameter inclusion distribution; (**b**) chord grain distribution.

Grain refinement was measured using a linear method, determining the frequency of specific chord groups on the microsection. The addition of REEs reduced the frequency of grains with chord lengths exceeding 40 µm, thus increasing the proportion of grains with shorter chord lengths (Figure 3a). A significant effect of the modification on the microstructure was observed in the shape and size of inclusions. Their diameter was used to assess their size. The modification resulted in the removal of large inclusions with diameters greater than 5.5 µm from the microstructure (Figure 3b). It should be emphasized that even a low frequency of large inclusions adversely affects the material’s plasticity. Heat treatment after casting included [22,23] normalization—940 °C, 1 h and tempering—710 °C, 2 h.

As a result of the modification of steel, the volume fraction, density of inclusions, and pearlite volume fraction decreased (Table 2 and Table 3).

### 3.1. Strength Characteristics

**Figure 4:**
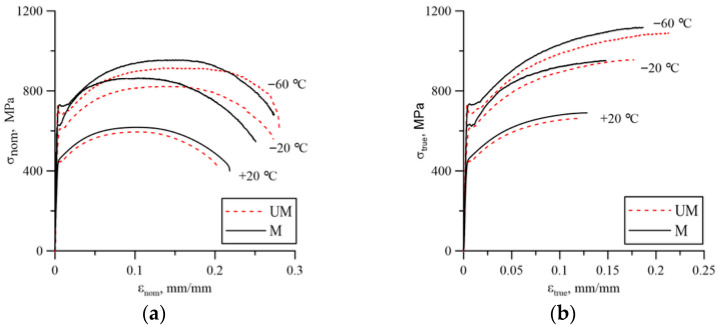
Stress–strain curves for individual temperatures—cast steel G17CrMo5-5: (**a**) nominal; (**b**) true.

The analyzed cast steel, regardless of its microstructure, exhibited a significant effect of test temperature on the yield strength and ultimate strength (Figure 4, Table 4). Lowering the temperature increased the strength characteristics as well as the strain corresponding to ultimate strength and fracture moment.

Changes in the microstructure of cast steel caused by the addition of rare earth elements slightly improved the strength characteristics (yield point—σ_0_, ultimate strength—σ_UTS_) throughout the temperature range of the tests.

### 3.2. Fracture Toughness Based on ASTM E 1820

To determine the fracture toughness for the ductile fracture mechanism, the *J*_R_ curve was used, while for brittle fracture, the relation 1 was used [18]:

(1) $$J_{c}=\frac{2A_{c}}{B(W-a_{0})}=\frac{2(A_{spr}+A_{pl})}{B(W-a_{0})},$$

where *A_C_* is the strain energy at the moment of fracture, calculated from the P(u) diagram, *A_C_* = *A_spr_* + *A_pl_*, *B* is the specimen thickness, *W* is the specimen width, and *a*_0_ is the fatigue crack length.

**Figure 5:**
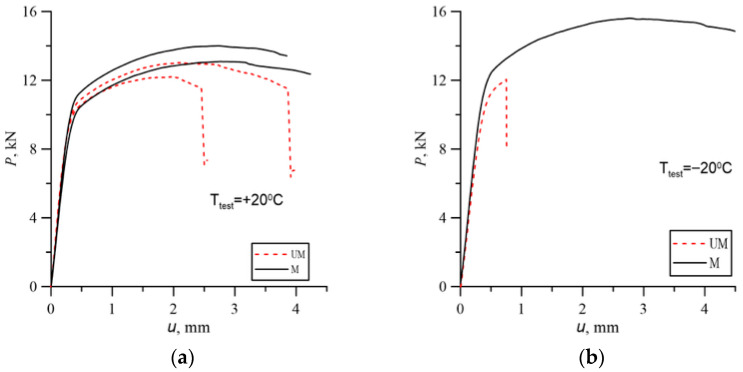
Example load charts of SENB specimens for test temperatures: (**a**) +20 °C; (**b**) −20 °C.

**Figure 6:**
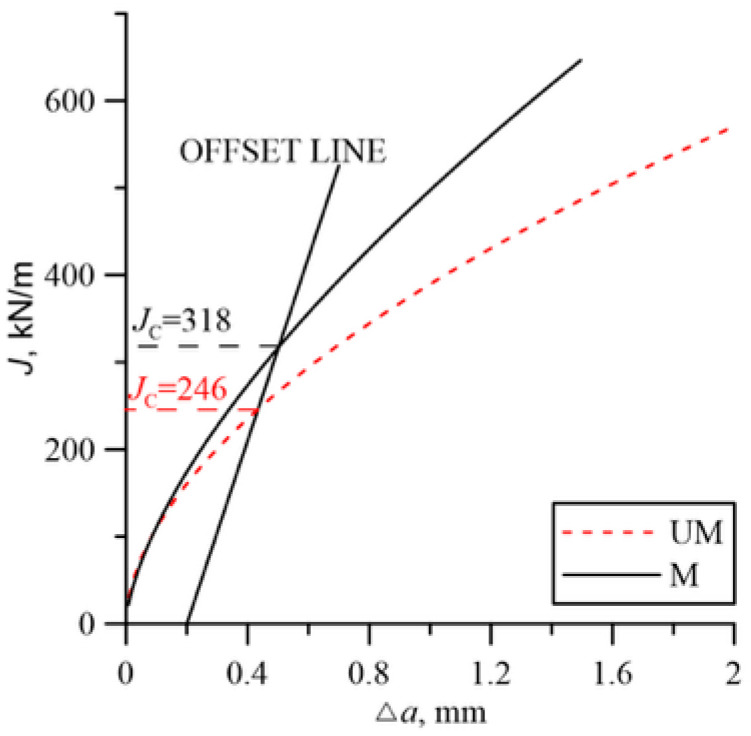
Example *J*_R_ curves for the tested cast steels at a test temperature of +20 °C.

Example load charts for SENB specimens are shown in Figure 5. At room temperature for unmodified cast steel, despite the occurrence of brittle fracture at the later stage of loading, the ductile growth of the crack was large enough to enable the plotting of the *J*_R_ curve (Figure 6).

**Figure 7:**
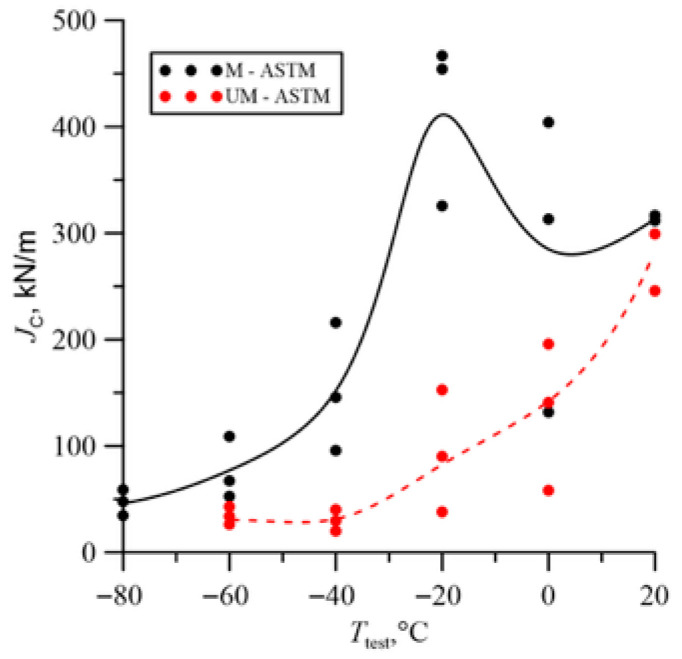
Fracture toughness at different test temperatures.

The fracture toughness results presented in Figure 7 indicate the beneficial effect of the modification. The fracture toughness of cast steel M was higher throughout the temperature range of the tests. The significant increase in fracture toughness of cast steel M at −20 °C was due to changes in the microstructure, especially inclusions. The matrix structure was similar (Table 3, Figure 1). The fracture toughness of the matrix was at a similar level, as evidenced by the results obtained at room temperature, where a ductile fracture mechanism occurred. Lowering the temperature to −20 °C resulted in brittle fracture of the inclusions. Large inclusions, which should be considered crack initiators, were more common in cast steel UM. Irregular shapes and clusters of inclusions additionally increased the local stress level at the interface with the matrix (Figure 2). In the case of the REE modification, the inclusions were globular and homogeneous in the material volume. The volume fraction decreased significantly from 2.07% to 0.33% (Table 2). The size and frequency of large inclusions was significantly smaller. For cast steel M, no inclusions with a diameter above 5.5 µm occurred, while for cast steel UM, the frequency of larger inclusions was about 20% (Figure 3a).

A large dispersion of fracture toughness results was obtained, especially during the fracture mechanism change interval (Table 5). Large discrepancies in fracture toughness values are commonly observed in steels and cast irons. In cast steel, the cause is the brittle fracture of inclusion particles at low temperatures, which are randomly distributed ahead of the crack front. Increasing the number of tested specimens would be advisable, but their number was limited by the ingot volume.

### 3.3. Microscopic Examination of Fracture Surfaces

**Figure 8:**
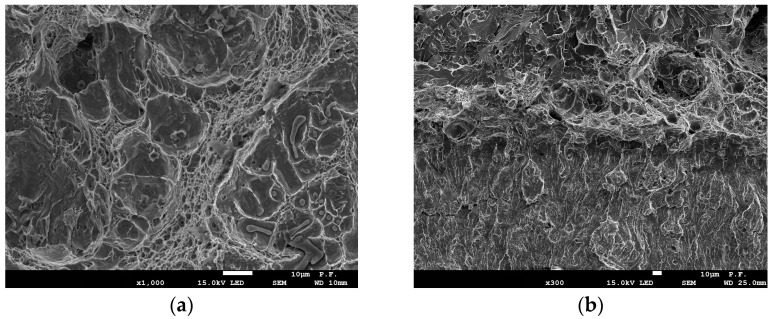
(**a**) Fracture surface of subcritical crack extension of UM cast steel at test temperature +20 °C: UM [24]; (**b**) fracture surface of subcritical crack extension of UM cast steel specimen at test temperature −20 °C.

For UM cast steel at room temperature, the dominant crack growth mechanism was ductile cracking. After a crack growth of approximately 0.9 mm, transcrystalline brittle cracking occurred (Figure 8a). However, at temperatures T = 0 °C and T = −20 °C, a mixed subcritical crack development occurred—a strip exhibiting a ductile propagation mechanism forms directly from the fatigue crack, and then the crack development mode changes to brittle. The cracking process initiated with particles approximately 4 µm in size (Figure 8a). The width of the ductile crack growth zone was small. The average dimensions were 128 µm for T = 0 °C and 116 µm for T = −20 °C. A further temperature decrease enabled brittle cracking for all specimens. The cracking process initiated with smaller inclusion particles of approximately 2 µm, which had the largest share (Figure 3).

**Figure 9:**
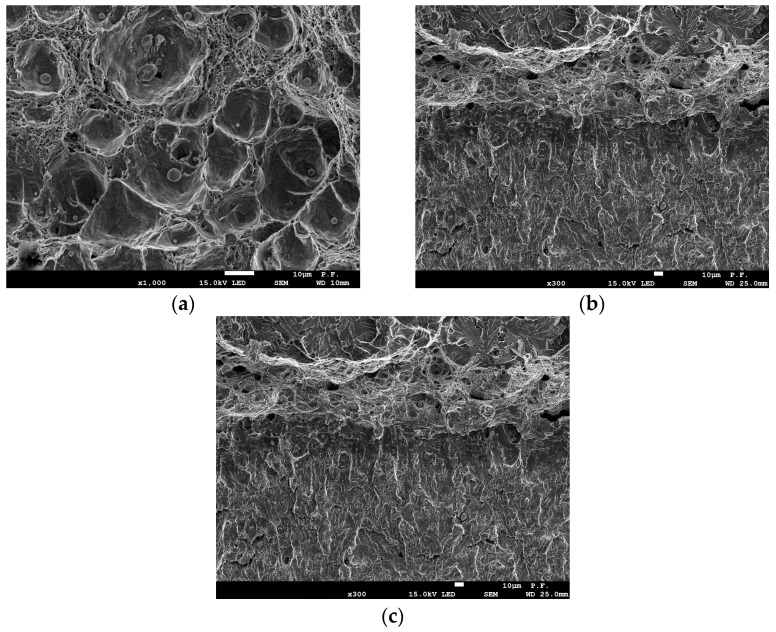
(**a**) Fracture surface of subcritical crack extension of M cast steel at test temperature +20 °C [24]; (**b**) fracture surface of subcritical crack extension of M cast steel specimen at test temperature −40 °C; (**c**) fracture surface of subcritical crack extension of M cast steel specimen at test temperature −60 °C.

Specimens made of modified cast steel fractured ductilely over the entire load range at temperatures from −20 °C to +20 °C. Cracking occurred through nucleation and the growth of voids around large inclusion particles (Figure 9a). A further temperature decrease enabled brittle fracture, which was preceded by a slight ductile growth. The ductile growth rate decreased with decreasing test temperature. At fractures in specimens tested at −60 °C, a ductile growth of approximately 100 µm was observed. Lowering the temperature to −80 °C enabled brittle fracture to occur in all tested specimens.

### 3.4. Fracture Toughness Based on Fractographic Analysis

**Figure 10:**
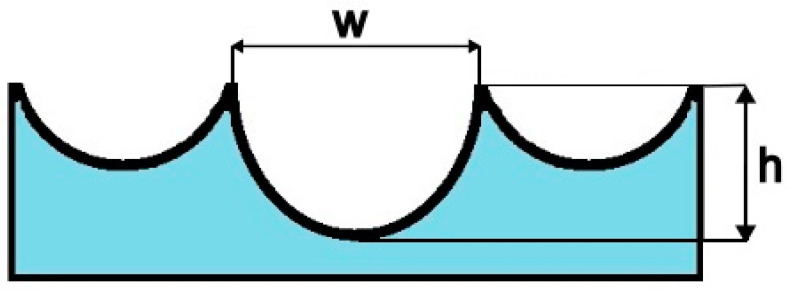
Schematic representation of the definition of fracture surface roughness, *m*.

The literature contains papers on the possibilities of fractographic analysis not only in a descriptive but also quantitative form [25]. This paper describes the proposal by R.O. Ritchie and A.W. Thompson for determining fracture toughness based on the analysis of fracture surface microroughness *m* [17]. This is defined as *m = h/w*, i.e., the ratio of the width to the height of a single void. This is schematically shown in Figure 10. It should be noted that the fracture toughness characteristic proposed by the authors is designated *J*_IC_. The use of the *J*_IC_ designation for fracture toughness characteristics obtained from geometric measurements of fracture surfaces is incorrect, hence the introduction of the *J*_M_ designation. The model presented in the cited publication [17] should be considered a local approach to fracture toughness assessment because it relies on microscopic examinations performed at the crack tip. This is due to the defined microroughness value *m = h/w*, where microroughness is defined based on the void created at the inclusion and generated during ductile crack development. Current technological advances in measuring devices dedicated to surface geometric structure (SGS) analysis enable the acquisition of high-resolution and precise data.

The critical value of the *J* integral at the moment of crack initiation also depends on the material microstructure:

(2) $$J_{M}\sim \frac{\sigma_{0}}{3}\ln(\frac{m^{2}}{3f_{p}})l_{0}^{*},$$

The basic assumption of the described approach is that the ratio of the height *h* to the diameter *D_p_* of the particle on which the void initiates is a measure of the local critical strain causing local failure, and is expressed as:

(3) $${\bar{\varepsilon }}_{f}^{*}\approx \ln\left(h/D_{p}\right),$$

or in terms of microroughness *m* and volume fraction of particles *f_p_* on which voids are initiated, is presented as:

(4) $${\bar{\varepsilon }}_{f}^{*}\approx \frac{1}{3}\ln\left(m^{2}/{3f}_{p}\right),$$

The above-mentioned formulas allow us to conclude that fracture toughness values can also be determined based on fracture surface tests, determining its characteristic features—the diameters and depths of voids.

The presented model should be considered a local approach to fracture toughness assessment because it relies on microscopic examinations performed at the crack tip. A limitation of this approach is the requirement for a ductile fracture mechanism.

The values h and w specified in the microroughness definition assumptions were compared with the defined values included in the standard [26]. According to the PN-EN ISO 4287 standard [26], the most similar value corresponding to the definition of the width of a single void is the average width of the grooves of the profile elements—*RSm*. The parameter that can be used to define the height h is the total profile height—*Rt*. This value is defined as the sum of the height of the highest profile peak *Zp* and the greatest depth of the profile recess *Zv* within the measuring section [26].

**Figure 11:**
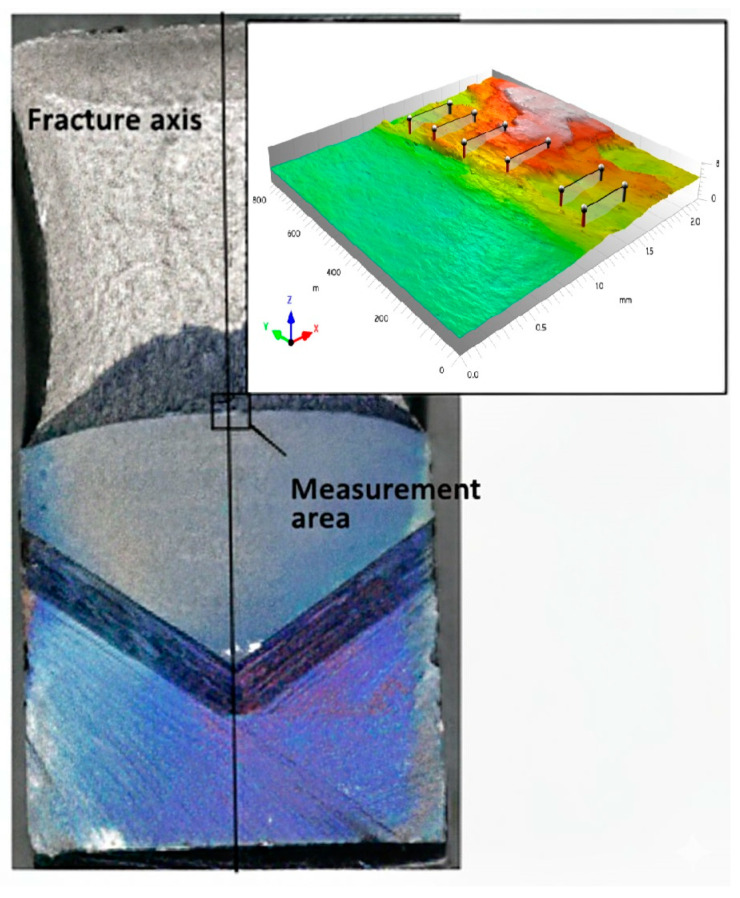
Macro view of the fracture surface with the microroughness measurement area carried and the 3D view.

To perform calculations using Formula (2), it is necessary to determine the area fraction of crack-initiating inclusions, *f_p_*. To estimate this value, measurements were taken of the diameter of voids or particles physically remaining on the fracture surface. Diameter measurements were taken in close proximity to the subcritical crack front. This approach to measurements was dictated by the inclusions or particles initiating the cracking process. Measurements conducted in this manner allowed for obtaining the surface area of inclusions in relation to the ductile fracture area. The growth area was assumed to be approximately the area of a rectangle whose sides corresponded to the width of the SEM image and the length of the ductile growth area estimated from fractographic analysis in cases of limited growth (Figure 11).

For specimens where significant ductility increases were observed, the dimensions were limited to the range of 0.1 mm to 0.5 mm in 0.1 mm increments.

**Figure 12:**
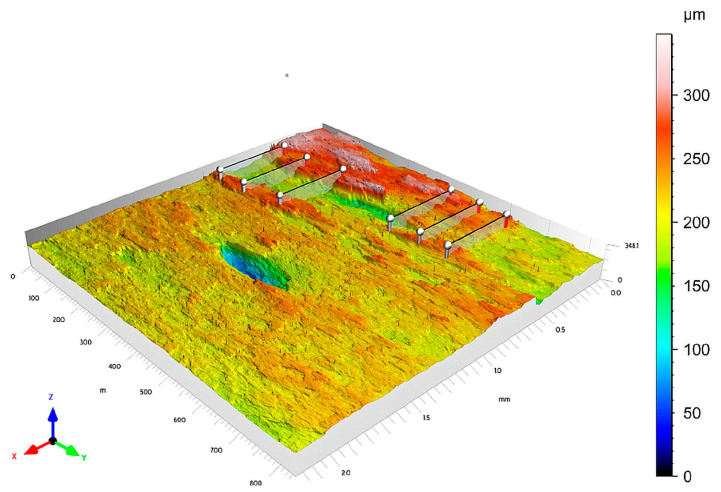
View of an example measurement area of a fracture made of cast steel G17CrMo5-5 without REEs for T = 20 °C with the location marked with profiles.

Measurements of selected surface roughness profile parameters were performed on fractured SENB specimens. The measurement area was located in the central part of the fractures, dominated by a plain strain. The measurement procedure was conducted under fixed, unchanging observation conditions for all operating parameters. A Taylor Hobson Talysurf CCI Lite measurement system was used for data collection. For the analyzed functions, a 20× magnification add-on was used. The measurement resolution set determined the pixel size in the lateral range (X–Y), adjustable at 0.85 × 0.85 μm. The analyzed measurement area, in the form of a 3D image measuring 0.8 mm × 2.0 mm, included the fatigue fracture zone, the stretch zone, and the crack growth zone. A schematic procedure is shown in Figure 12. Six roughness profiles were generated on the resulting 3D surface view.

Measurement sections for fractures where a significant plastic growth was observed were generated every 0.1 mm, ranging from 0.1 mm to 0.5 mm. For cases where the plastic growth length of the crack was less than 0.5 mm, the length of the measurement section corresponded to the growth length. Based on the roughness profiles, the following parameters were determined using Mountains 8 software: *R_Sm_* and *R_t_*, which were used to determine the *M* factor.

Tests for UM cast steel were conducted on breakthroughs with a test temperature range of +20 °C to −20 °C, where plastic crack growth was observed. Surface geometric structure analyses for M cast steel were conducted on fractures with a test temperature range of +20 °C to −60 °C. When testing the fracture surfaces of cast steel, we face the challenge of locating profiles to obtain numerical data for the assumed parameters required for further analysis. Figure 12 presents a 3D image of a fracture with a test temperature of +20 °C, with linear profiles plotted on it. These types of problems, resulting from fracture structure, make it impossible to provide a universal location for the profiles. The results of the obtained measurements of surface roughness parameters are presented below, organized in Table 6 and Table 7 together with the calculated standard deviation (SD).

**Figure 13:**
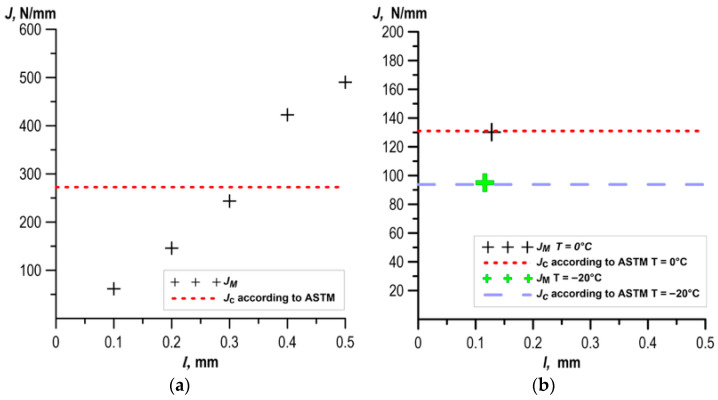
Comparison of fracture toughness values determined according to the ASTM method and based on fracture roughness measurements of UM cast steel: (**a**) T = +20 °C; (**b**) T = 0 °C and T = −20 °C.

The results of the calculated fracture toughness values for specimens made of UM cast steel are shown in Figure 13. Profile roughness parameters were determined on sections with lengths corresponding to the width of the ductile crack growth zone. For a fully ductile subcritical crack growth, roughness parameters were determined on sections *l*: 0.1 mm, 0.2 mm, 0.3 mm, 0.4 mm, and 0.5 mm.

The fractures for the M cast steel specimens tested at temperatures from −20 °C to +20 °C were ductile, therefore the same roughness measurement methodology was used as for the UM cast steel tested at +20 °C. However, at temperatures T = −40 °C and T = −60 °C, a mixed pattern of subcritical crack development occurred. Roughness profile measurements were performed in the ductile growth zone, the average dimensions of which were 140 µm for T = −40 °C and 100 µm for T = −60 °C.

**Figure 14:**
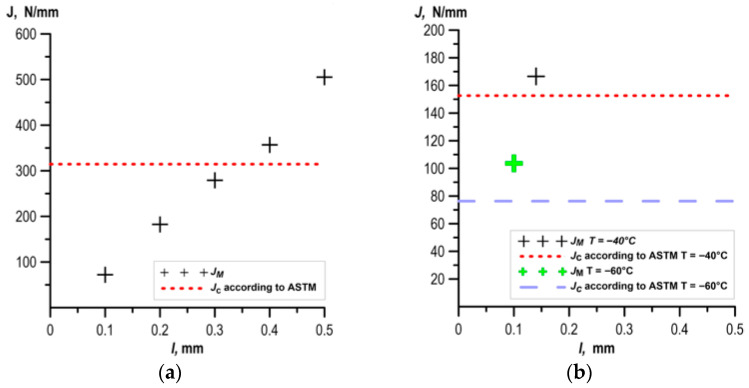
Comparison of fracture toughness values determined according to the ASTM method and based on fracture roughness measurements of cast steel breakthrough M: (**a**) T = +20 °C; (**b**) T = −40 °C and T = −60 °C.

The results of the fracture toughness calculations obtained from the fracture surface roughness measurements of specimens for cast steel M are presented in Figure 14.

A comparison of fracture toughness values determined according to the ASTM recommendations and based on roughness measurements is presented in Figure 13 and Figure 14. Comparing the results presented in the figures, it can be seen that for the case of a fully ductile increment, as the length of the measurement section l increased, the *J*_M_ value also increased. In the case of short ductile increments, the *J*_M_ values were determined based on roughness parameters determined from profiles measured on sections of appropriate length.

**Figure 15:**
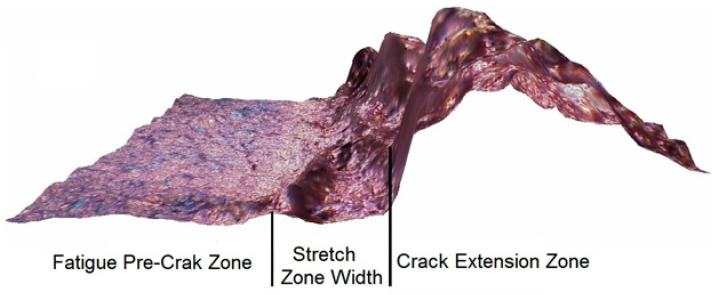
Surface profile of the SENB specimen breakthrough [27].

Due to the limitation of the fracture toughness method based on roughness measurements to cases where only ductile crack growth occurs, a method based on measuring the stretch zone width was used. In the case of brittle fracture without subcritical crack growth but only after material yielding before the crack tip, the *J*_IC_ (or *J*_C_) and *J*_i_ values are comparable, hence the decision to use this method. A modified method for measuring the stretch zone width was used in this paper. The study relied on the use of a scanning electron microscope (SEM) to measure the stretch zone width (SZW) at fractures in SENB specimens. This zone is visible as a rounded transition between the fatigue crack and the subcritical crack (Figure 15). Measurements were taken at least three places in the central part of the breakthrough.

**Figure 16:**
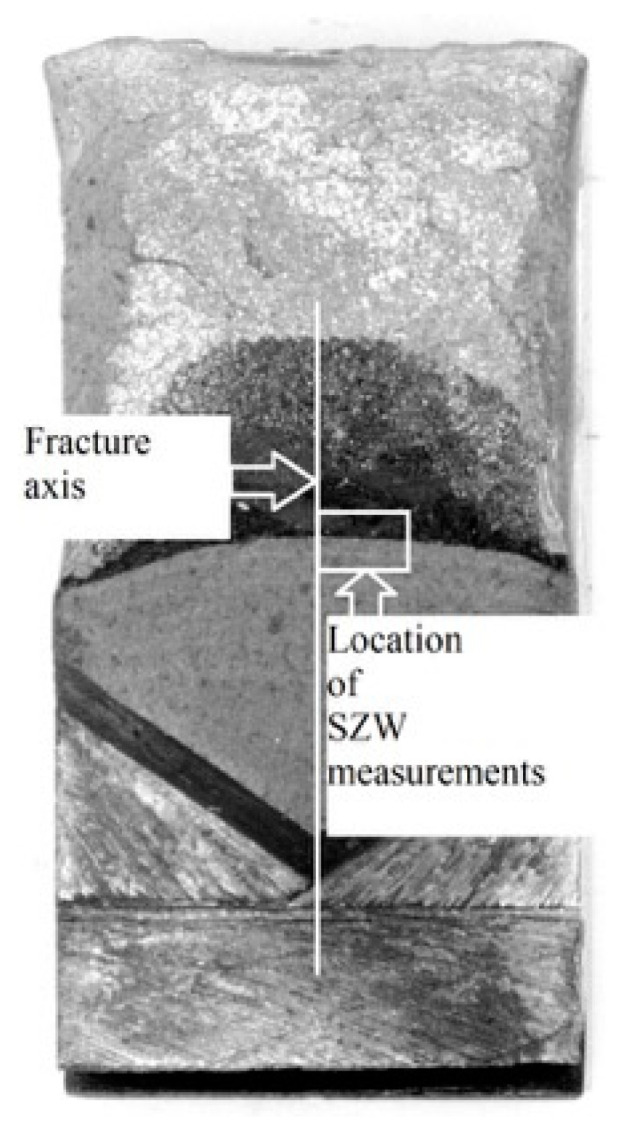
Scheme of performing SZW measurements at the breakthrough.

This required precise positioning of the specimen under the microscope: the cross-sectional axis had to align with the edge of the image (Figure 16) at 40× magnification. The operator then moved the microscope stage to position subsequent measurement points using XYZ coordinates. Scanning electron microscopy (SEM) images were used to determine the width of the blunting zone. Observations of all analyzed specimens were performed while maintaining constant, uniform recording parameters. The applied magnification was 750×, and the working distance (WD) was maintained at 25 mm. Furthermore, data were recorded at a constant accelerating voltage (E0) of 15 kV.

Traditional methods for measuring the width of the stretch zone (SZW) are inaccurate because they rely on measurements at selected, single points. Due to the irregular structure of this zone, this approach does not reflect its actual characteristics [28,29]. Therefore, a new approach based on image analysis was introduced. Instead of measuring the width at points, the surface area of the stretch zone was measured in a specific fracture area. Specialized image analysis software, e.g., ImageJ2x, was used for this purpose. The key element of the method is the transition from measured surface area to average width. Knowing the width of the measurement area, which remains constant for a given set of SEM observation parameters (constant at a given magnification and working distance), we can calculate the average value of the stretch zone width. This approach provides an averaged zone width for the entire analyzed area, which is a more comprehensive indicator than measuring individual, discrete points. The ImageJ2x software was calibrated according to the instructions based on SEM images taken with identical parameters, resulting in a constant pixel-to-length ratio of 8.06 pixels/µm for the applied 750× magnification.

On SEM images of fractures of the UM and M cast steel specimens, measurements of the stretch zone width (${∆\bar{a}}_{SZW}$) were made using image analysis for specimens from the test temperatures where only brittle fracture was observed (Table 8). Cast steel is a representative example of a material in which the SZW measurement is difficult. In this material, especially for the cast steel without the REE additive, local disappearances of the stretch zone occurred. Table 8 presents the value representing the average width of the stretch zone based on nine measurements of the stretch zone surface areas (three measurements in the range of 0–900 µm on each of the three specimens) (Figure 16). The selection of the measurement range for SZW was made based on the experimental studies described in the article [30].

**Figure 17:**
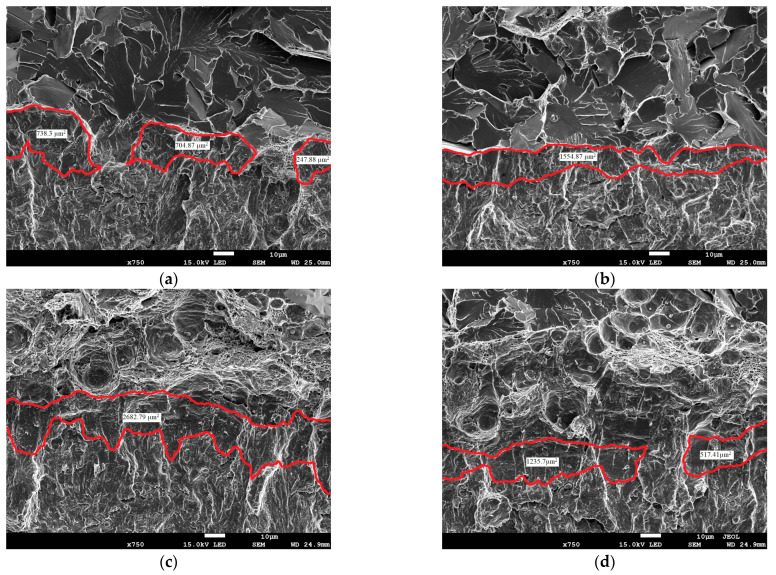
View of the course of the stretch zone of cast steel for T = −60 °C from the fracture center: (**a**) 300 µm^2^ for UM; (**b**) 900 µm for UM; (**c**) 300 µm for M; (**d**) 900 µm for M.

Example SEM images of fracture surfaces of the tested specimens, subjected to image analysis, with the stretch zone areas marked, are presented below in Figure 17. These zone disappearances were caused by inclusion particles located just near the crack front, initiating cracking. In the UM cast steel, complete stretch zone disappearances could be observed at several measurement locations, dividing it into two areas. The presence of inclusions clearly affected the average width of the stretch zone, which translated not only into numerical values but also its shape and course before the crack front. M cast steels performed best in this case, with comparable values achieved only at a test temperature of −60 °C (Figure 17) (Table 8).

The experimental studies clearly indicate that modifying the material’s microstructure has a significant and beneficial effect on the width of the stretch zone ahead of the crack front. Analysis of the mean values of the stretch zone width ($∆{\bar{a}}_{SZW}$) confirms the thesis that modified steels (M) are characterized by the highest efficiency of the blunting mechanism. While the mean stretch zone width for the unmodified material (UM) ranged from 8.21 µm (at −60 °C) to 8.82 µm (at −40 °C), the modified material (M) reached an average value of 10.61 µm (at −80 °C), which represents an increase of approximately 20–30% (Table 9). This significant increase in the stretch zone width is direct evidence that the modification improved the material’s ability to dissipate fracture energy. Moreover, higher values ($∆{\bar{a}}_{SZW}$) for material M were obtained with a satisfactory level of repeatability of the results (low scatter SD = 1.28 μm), which indicates a stable and effective change of the microstructure.

As a result of preliminary research conducted by the authors [31,32], the formula proposed by Shih (Formula (5)) was chosen to calculate *J*_i_.

(5) $$\;J_{i}=\frac{2\sigma_{0}∆{\bar{a}}_{SZW}}{d_{n}},$$

This proposal is the most universal of those proposed in the literature [33,34,35,36].The *d_n_* coefficient necessary for the calculations was determined according to Guo’s proposal [37]. Calculations of the fracture toughness value at the moment of initiation *J*_i_ were performed based on the average values determined in the appropriate measurement section.

Similar values of *J*_i_ and *J*_IC_ (or J_C_) were recorded at test temperatures from −40 °C for UM cast steel, and at −80 °C for M cast steel at test temperatures concerning the dominance of brittle fracture [31,38].

### 3.5. Numerical Results of Stress Distribution Before the Crack Tip

To assess the effect of particle shape on stress fields in front of the crack, we used results obtained from specimens of G17CrMo5 5 cast steel, unmodified and modified with rare-earth metals. Modification caused a number of changes in the cast steel—it homogenized the microstructure and significantly changed the shape of the inclusion particles from irregular to globular (Figure 2). These microstructural changes, in turn, led to a significant increase in fracture toughness in the modified cast steel. Based on the experimentally tested specimens, numerical calculations were performed to determine the stress distributions in front of the crack tip.

**Figure 18:**
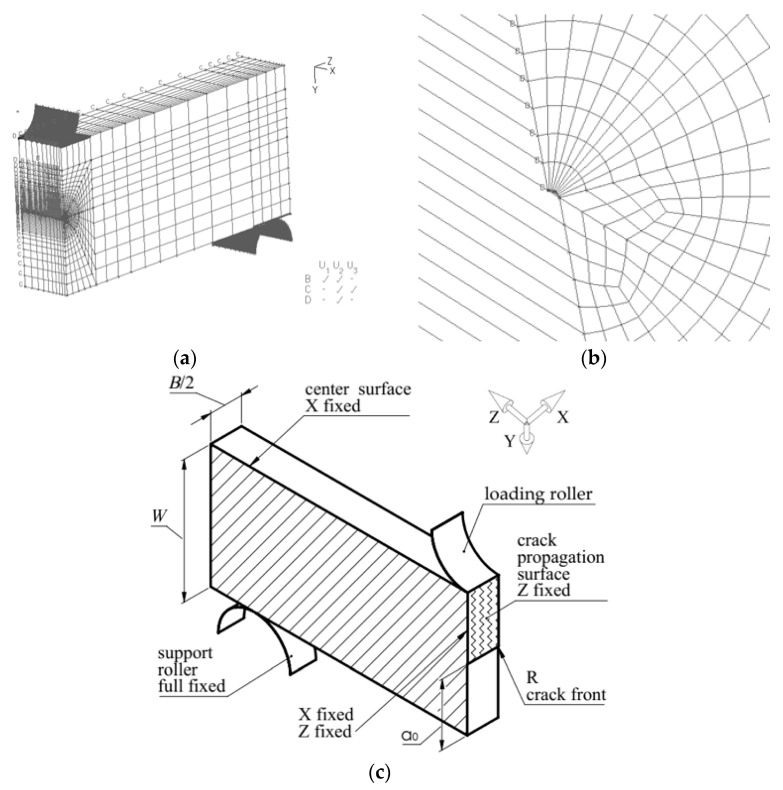
Model of the SENB specimen used in numerical calculations: (**a**) view with boundary conditions; (**b**) arc-shaped crack tip; (**c**) scheme of boundary conditions and loading.

Calculations were performed in ABAQUS version 6.12-2. Due to the existing symmetry, a quarter of the three-dimensional SENB specimen was modeled (Figure 18a). The application of appropriate boundary conditions reduced the calculation time. The boundary condition definitions prevented the displacement of the specimen surface common to the crack front in the Z direction, the surface passing through the center of the specimen thickness in the X direction, and the supporting roller was completely immobilized (Figure 18c). The line formed at the intersection of two mutually perpendicular symmetry planes of the specimen was allowed to move only in the Y direction. The specimen was divided into layers in the thickness direction, with the distance between the layers becoming denser toward the free surface. This densification resulted from larger changes in the stress components at the free surface. The crack tip was represented as a quarter circle with a radius determined based on the effective stress and strain profiles before the crack tip (Figure 18b).

C3D8R elements were used in the calculations. The finite element mesh size was not a constant, but depended on the stress–strain relationship and the applied load. To ensure adequate mesh quality, the built-in ABAQUS Mesh Verify tool was used. Additionally, the convergence of the obtained fracture toughness values and stress distributions was verified in subsequent iterations by refining the finite element mesh toward the crack tip. If the difference in fracture toughness values and the maximum cracking component did not exceed 3%, the mesh size was considered correct.

Load was defined by the displacement of the upper loading roller with a deflection corresponding to that recorded during the experiment. The onset of crack growth in brittle fracture was determined based on the maximum force value and the corresponding displacement of the load point. For ductile fracture, it was determined based on the compliance-COD diagram. A representative sample giving a fracture toughness result close to the average value was selected for the calculations. The material definition procedure used actual stress–strain diagrams derived from diagrams obtained during uniaxial tensile tests and calibrated according to the recommendations described in the articles [38,39,40,41].

**Figure 19:**
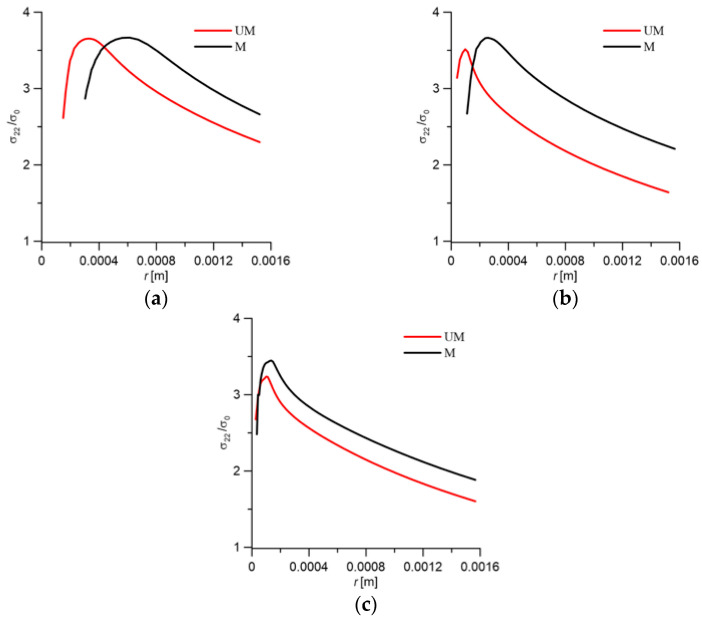
(**a**) Normal stress distributions σ_22_ for unmodified and modified G17CrMo5 5 cast steel at the test temperatures: (**a**) 20 °C; (**b**) −20 °C; (**c**) −60 °C.

Figure 19 shows the nature of the change in the stress tensor components in the direction of crack growth (*r* is the distance from the crack tip). The graphs represent data for the middle layer of the specimen, aligned with its longitudinal symmetry plane. Stresses normal to the crack plane σ_22_ play a crucial role in the analysis of brittle cleavage fracture. Depending on the maximum values, particularly σ_22_/σ_0_, and the location of the maximum, conclusions can be drawn about the fracture mode—brittle, mixed, or ductile [42,43].

Example graphs of stress distributions normal to the crack plane σ_22_ at various temperatures (20 °C, −20 °C, and −60 °C) are shown in Figure 19.

The nature of the σ_22_ stress distributions allows us to conclude:

- For modified cast steel, stresses at each of these temperatures were higher than for the unmodified material;
- The location of the maximum values of the σ_22_ stress distributions for M was slightly further from the crack tip than for UM;
- The σ_22_ stress diagrams with M were characterized by a wider outline around the maximum values.

These characteristics of the diagrams indicate higher fracture toughness for the M material compared to UM.

## 4. Discussion

Carrying out procedures aimed at changing the microstructure features (i.e., fragmentation, homogenization of grain size, and changing the size and shape of inclusions), homogenization in the volume of the material had a positive effect on the strength characteristics and fracture resistance.

The changes in the obtained fracture toughness values at room temperature were insignificant. The largest increase was observed in the transition region of the ductile-to-brittle fracture mechanism. The main crack initiators were large non-metallic inclusions, which, in the temperature range from −40 °C to 0 °C, were characterized by greater brittleness relative to the matrix. The REE modification reduced the frequency of large inclusions and changed the shape to spheroidal. The smaller size and spheroidal shape of the inclusions lowered the stress concentration level in the inclusion region, allowing the ferritic-bainitic matrix to undergo plastic deformation. The increase in plastic deformation enabled for the absorption of significant amounts of energy during loading, resulting in increased fracture toughness. Lowering the test temperature to −60 °C and −80 °C resulted in a transition to the brittle fracture region for the analyzed melts. In this area, a beneficial, but slight, effect of microstructure on fracture toughness was observed, as crack initiation begins with small inclusions. Furthermore, the matrix became more brittle, reaching the level of non-metallic inclusions.

Applying a fracture toughness assessment method based on fracture surface roughness measurements allowed for fracture toughness levels comparable to the critical value of the *J*–*J*_IC_ integral for the case of subcritical ductile crack growth in the specimen. Unfortunately, satisfactory results were not achieved for the case of a fully brittle (cleavage) fracture mechanism.

In the case of fully ductile crack growth, we obtained good agreement with the results obtained with the ASTM method. In the case of fully ductile fracture tests of specimens made of UM cast steel, a similar level of fracture toughness was obtained at T = +20 °C for a section with a length of *l* = 350 µm (Figure 13a). For M cast steel, a similar level of fracture toughness was obtained: at T = +20 °C for a section with a length of *l* = 400 µm; at T = 0 °C for a section with a length of *l* = 350 µm; at T = −20 °C for a section with a length of *l* = 350 µm (Figure 14a).

**Figure 20:**
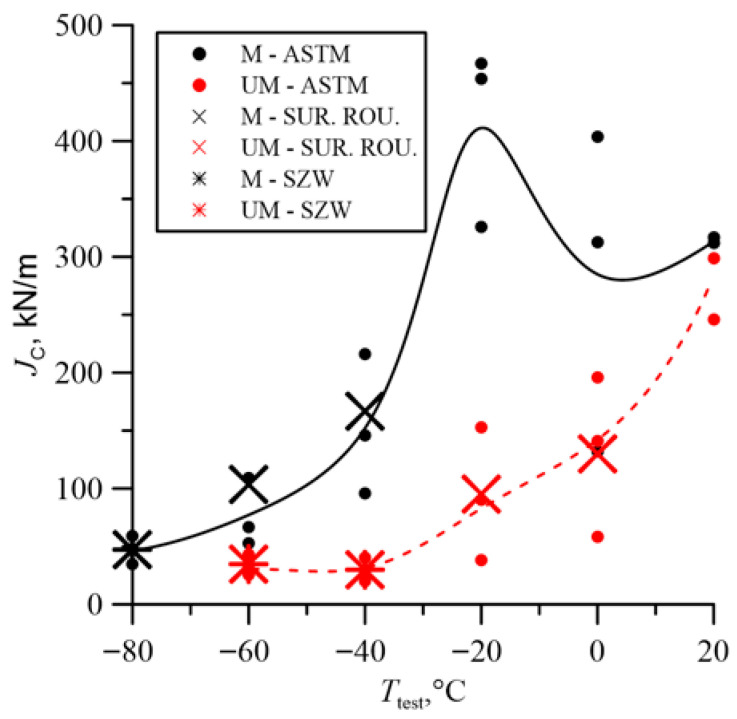
Comparison of fracture toughness obtained according to ASTM E1820 and based on roughness and SZW measurements.

For the case of limited ductile subcritical crack growth (up to 0.14 mm), the geometric surface dimensions were determined over the length of section l corresponding to the ductile crack growth value. The results presented in Figure 13a,b for cast steel UM and Figure 14a,b for cast steel M demonstrated good agreement between the fracture toughness values obtained from roughness measurements and the ASTM E1820 method (Figure 20).

Changing the shape and size of inclusions, as well as homogenizing the microstructure, favorably affected the stress field distributions in front of the crack. The presented σ_22_ stress distributions primarily determined the fracture process. For cast steel M, at each of the numerically analyzed test temperatures, the range of near-maximum stresses ahead of the crack front was wider. The greatest differences occurred at the temperature at which the differences in fracture toughness were greatest, i.e., −20 °C. The location of the maximum σ_22_ stress was always further from the crack front for the modified cast steel; however, as the temperature decreased, these locations became closer together.

## 5. Conclusions

Modification of REE cast steel is an effective method for:

- Improving microstructural properties, such as size homogenization, dispersion, and spheroidization of inclusions;
- Increasing fracture toughness, especially in the context of a change in the fracture mechanism from ductile to brittle, preceded by plastic growth;
- Favorable stress field distribution ahead of the crack front, particularly in the temperature region of greatest increase in fracture toughness;
- Improving strength characteristics, but not to the same extent as fracture toughness.

Fractographic analysis is emerging as a promising approach to quantitatively assessing fracture toughness. It will be worthwhile expanding this approach and testing it on a broader range of structural materials. The type of measurement method used depends on the fracture mode:

- In the case of ductile subcritical crack growth, a method based on measurements of fracture surface roughness parameters should be used;
- In the case of brittle fracture without subcritical crack growth but only after the material has plasticized before the crack tip, a method based on measurements of the stretch zone surface area should be used.

## Figures and Tables

**Table 1:** Chemical composition of cast steel G17CrMo5-5 (in % mass) [21].

	Contents [%]								
	C	Si	Mn	Cr	Mo	Ni	Al	S	P
acc PN-EN 10293	0.15 – 0.20	max 0.6	0.50 – 1.00	1.00 – 1.50	0.45 – 0.65	-	-	max 0.020	max 0.025
UM	0.18	0.4	0.9	1.2	0.53	0.07	0.041	0.015	0.022
M	0.16	0.37	0.62	1.22	0.53	0.12	0.050	0.013	0.022

**Table 2:** Stereological parameters of non-metallic inclusions.

	Volume Fraction	Inclusions Density, N_A_
	%	mm^−2^
UM	2.07	1969
M	0.33	1128

**Table 3:** Stereological parameters of the cast steel microstructure.

	Average Chord	Pearlite Volume Fraction
	μm	%
UM	35.1	23.1
M	24.0	20.1

**Table 4:** Mechanical properties of G17CrMo5 cast steel (nominal values).

T	σ_0_	SD	σ_UTS_	SD	Elongation
°C	MPa	MPa	MPa	MPa	%
UM					
20	444; 442; 441	1.5	587; 595; 596	4.9	20
−20	592; 622	21.2	824; 832	5.7	24
−40	641	-	897	-	26
−60	726; 704	15.6	916; 912	2.8	27
M					
20	430; 457; 468	19.5	611; 618; 620	4.7	22
−20	630; 628	1.4	865; 839	18.4	25
−60	698; 728	21.2	1081; 955	89.1	27
−80	747	-	970		26

**Table 5:** Fracture toughness of G17CrMo5 cast steel.

T	*J*_C_	SD
°C	kN/m	kN/m
UM		
20	299; 246	37.5
0	141; 58; 196	69.5
−20	90; 38; 153	57.6
−40	30; 20; 40	10
−60	26; 34; 43	8.5
M		
20	317; 312	3.5
0	132; 313; 404	138.5
−20	326; 467; 454	77.9
−40	96; 216; 146	60.3
−60	53; 67; 109	29.1
−80	59; 48; 35	12.0

**Table 6:** Averaged parameter measurement results *R_Sm_ i R_t_* for UM cast steel.

T °C	*l* µm	*R_t_* µm	SD µm	*R_Sm_* µm	SD µm
+20	100	16.15	7.0	38.16	10.2
	200	22.88	8.9	52.99	10.3
	300	23.03	10.0	49.69	9.7
	400	31.74	6.4	35.39	8.8
	500	33.90	8.6	54.22	7.5
0	128	39.41	11.2	7.88	3.9
−20	116	15.96	7.8	23.50	8.7

**Table 7:** Averaged parameter measurement results *R_Sm_ i R_t_* for G17CrMo5-5 for M cast steel.

T °C	*l* µm	*R_t_* µm	SD µm	*R_Sm_* µm	SD µm
+20	100	20.76	7.6	36.57	16.5
	200	26.15	11.8	35.47	8.3
	300	30.77	9.1	48.12	11.9
	400	26.48	7.1	54.08	12.9
	500	37.60	6.4	52.64	8.4
0	100	16.39	5.2	43.17	13.7
	200	21.42	8.1	44.54	6.4
	300	26.66	9.7	56.86	10.1
	400	39.26	9.9	48.47	11.3
	500	39.26	8.3	47.22	10.7
−20	100	20.03	9.9	20.76	8.9
	200	25.41	10.4	59.61	14.4
	300	30.48	10.7	55.15	10.1
	400	32.06	6.7	49.85	7.9
	500	32.09	7.5	50.91	8.9
−40	140	25.43	3.7	10.35	4.6
−60	100	15.20	6.6	37.07	10.4

**Table 8:** Results for cast steel G17CrMo5-5 for the brittle fracture mechanism.

	T °C	TEST 1			TEST 2			TEST 3			${∆\bar{a}}_{SZW}$ µm	SD µm
UM	−40	6.46	6.32	9.65	9.61	9.94	7.65	10.25	9.78	9.68	8.82	2.93
	−60	9.68	8.09	7.52	9.17	8.96	7.88	9.23	6.15	7.20	8.21	1.15
M	−80	10.15	12.21	8.95	11.24	10.28	10.56	12.63	10.56	8.88	10.61	1.28

**Table 9:** Fracture toughness calculated *J*_i_ based on Equation (5).

G17CrMo5-5	T	*d*_n_	$∆{\bar{a}}_{SZW}$	*J*_i_	*J*_C_, *J*_IC_
	°C		μm	kN/m	kN/m
UM	−40	0.534	8.82	27.17	30.05
	−60	0.535	8.21	27.83	34.51
M	−80	0.515	10.61	40.62	47.40

## Data Availability

The original contributions presented in this study are included in the article. Further inquiries can be directed to the corresponding author.
